# Moth Communities Reveal High Stability Despite Ongoing Compositional Shifts Over Five Years Following Hurricane Disturbance

**DOI:** 10.1002/ece3.72278

**Published:** 2025-10-09

**Authors:** Aura M. Alonso‐Rodríguez, Pablo E. Gutiérrez‐Fonseca, Scott E. Miller, Taylor H. Ricketts

**Affiliations:** ^1^ Rubenstein School of Environment and Natural Resources University of Vermont Burlington Vermont USA; ^2^ Gund Institute for Environment University of Vermont Burlington Vermont USA; ^3^ Biology Department University of Puerto Rico at Mayagüez Mayagüez Puerto Rico; ^4^ Department of Entomology National Museum of Natural History, Smithsonian Institution Washington DC USA

**Keywords:** climate change, disturbance ecology, ecological stability, hurricanes, Lepidoptera, temporal dynamics

## Abstract

Extreme climatic events are expected to increase in frequency and severity under climate change, with lasting consequences for ecological communities worldwide. Global insect declines have raised concerns for biodiversity conservation and ecosystem stability, as shifts in insect communities can trigger cascading effects across trophic levels. Yet insect responses to large‐scale disturbances remain poorly understood, particularly in tropical forests where long‐term datasets are scarce and taxonomic knowledge is limited. We examined the response trajectories and stability of moth communities in two forest types in Puerto Rico following the September 2017 hurricanes Irma and María. Using monthly surveys conducted 5 months before and 6 months after the storms, followed by annual surveys over 5 years, we tracked changes in moth abundance, richness, and composition. We also evaluated ecological stability across multiple dimensions (i.e., resistance, resilience, recovery, and temporal stability) for the entire community and separately for Crambidae, Erebidae, and Geometridae. Despite initial declines, abundance and richness surpassed baseline levels within the first year, especially in old‐growth tabonuco stands, which may have provided more stable microhabitats and resources than palm stands. Resistance varied by family, with grass‐feeding crambids increasing in abundance and arboreal‐feeding geometrids experiencing the greatest species loss. Abundance and richness stabilized within 2 years, likely influenced by trophic interactions that regulated insect outbreaks. Species composition continued to shift over time, reflecting ongoing reassembly, while compositional and functional stability metrics suggested recovery within 5 years. This highlights both the resilience of the moth community and the dynamic nature of post‐disturbance reassembly. Our findings underscore the value of multi‐year, post‐disturbance datasets for uncovering recovery pathways and enhancing our understanding of ecological stability. As extreme events intensify across biomes, insights into resilience dynamics will be critical for sustaining insect biodiversity and the ecological functions they provide.

## Graphical abstract

We examined the temporal dynamics and ecological stability of tropical forest moth communities using a unique dataset spanning 6 months before and up to 5 years after two extreme hurricanes. While abundance and richness recovered within 2 years, species composition continued to shift, indicating ongoing reassembly with a directional trajectory toward recovery. These findings demonstrate the resilience of moth communities following extreme hurricanes, which are becoming more frequent under global change.

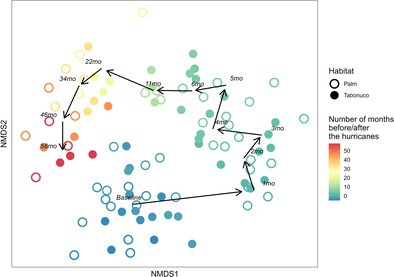

## RESUMEN

Los modelos climáticos predicen que los eventos extremos, como los huracanes, aumentarán en frecuencia y severidad debido al cambio climático, afectando a las comunidades ecológicas en todo el mundo. El declive global de los insectos ha generado preocupación por su impacto en la biodiversidad y estabilidad ecosistémica, ya que los cambios en sus comunidades pueden desencadenar efectos en la red trófica. No obstante, las respuestas de los insectos a grandes disturbios siguen siendo poco estudiadas, especialmente en bosques tropicales, donde escasean los datos a largo plazo y el conocimiento taxonómico es limitado. En este estudio, examinamos la respuesta y estabilidad de comunidades de polillas en dos tipos de bosque en Puerto Rico tras los huracanes Irma y María de septiembre de 2017. Con muestreos mensuales realizados 5 meses antes y 6 meses después de las tormentas, seguido de muestreos anuales durante 5 años, documentamos cambios en abundancia, riqueza y composición. Además, evaluamos la estabilidad ecológica a través de varias dimensiones (resistencia, resiliencia, recuperación y estabilidad temporal) para toda la comunidad y para las familias Crambidae, Erebidae y Geometridae por separado. Tras un descenso inicial, la abundancia y riqueza de polillas superaron los niveles de referencia durante el primer año, especialmente en bosques maduros de tabonuco, que pudieron haber brindado microhábitats y recursos más estables que los bosques de palma. La resistencia varió según la familia, con un aumento en la abundancia de crámbidos (que se alimentan de gramíneas) y una pérdida significativa de especies en geométridos (que se alimentan de vegetación arbórea). La abundancia y riqueza se estabilizaron en 2 años, posiblemente por interacciones tróficas que controlaron los brotes de herbívoros. La composición de especies siguió cambiando a través del tiempo, reflejando una comunidad en constante recambio, mientras que las métricas de estabilidad composicional y funcional sugieren que hubo recuperación dentro de un período de 5 años. Esto resalta tanto la resiliencia de la comunidad de polillas como el carácter dinámico del reensamblaje post‐disturbio. Nuestros hallazgos destacan la importancia de contar con datos multianuales post‐disturbio para revelar las trayectorias de recuperación y mejorar nuestro entendimiento de la estabilidad ecológica. A medida que los eventos extremos se intensifiquen en todos los biomas, entender estas dinámicas de resiliencia será clave para conservar la biodiversidad de insectos y las funciones ecológicas que estos desempeñan.

## 1 Introduction

Understanding how biological communities and ecosystem function respond to and recover from disturbances is a central ecological question in the Anthropocene (Turner and Seidl 2023), as human‐induced environmental changes drive unprecedented rates of biodiversity loss (Turvey and Crees 2019). Disturbances are abrupt events that can destabilize trophic dynamics and alter habitat conditions, creating opportunities for species reassembly and driving shifts in biodiversity and ecosystem functioning across wide spatial and temporal scales (Willig and Presley 2018; Bowd et al. 2021). Ecologists have long sought to understand how species assemblages reorganize after disturbance events (Connell and Slatyer 1977; Yih et al. 1991; Platt and Connell 2003), yet responses vary by disturbance characteristics, local species interactions, and the influence of past selection for life‐history traits associated with disturbance legacies (Johnstone et al. 2016; Seidl et al. 2022). In the context of accelerating global change, empirical studies that track community dynamics following disturbances are essential for refining ecological theory on post‐disturbance reassembly and guiding effective conservation strategies.

Disturbance regimes are expected to shift under a changing climate (Turner 2010), with high‐intensity hurricanes (category 4–5) projected to become more frequent (Knutson et al. 2020). These powerful storms have the capacity to reshape ecosystems, altering all aspects of their functioning and potentially causing long‐lasting effects on the composition and evolutionary trajectories of local biota (Lugo 2008; Xi 2015; Ibanez et al. 2019). To assess the ecological consequences of such disturbances and how they vary across taxa, the concept of ecological stability provides a valuable framework for evaluating post‐hurricane ecological dynamics (Donohue et al. 2016; Kéfi et al. 2019).

Stability can be assessed through multiple dimensions, including *resistance* (i.e., the ability of a system to withstand disturbance with minimal change), *resilience* (i.e., the speed of recovery after disturbance, sensu Pimm 1984), *recovery* (i.e., the extent to which a system returns to its pre‐disturbance state), and *temporal stability* (i.e., the degree of fluctuation in a system over time) (Hillebrand et al. 2018). Although ecosystems may not always return to a single equilibrium state and may instead transition to alternative stable states depending on disturbance magnitude (Holling 1973; Schröder et al. 2005), this framework allows for the comparison of community responses using pre‐disturbance baselines as mathematical points of reference (May 1972; Pimm 1984). Additionally, it enables the evaluation of both compositional stability (i.e., changes in community structure) and functional stability (i.e., changes in the ecosystem processes performed by the taxa of interest), providing a more comprehensive view of community assembly and the maintenance of ecosystem functions following disturbances (Oliver et al. 2015; Hillebrand and Kunze 2020).

While short‐term studies during early succession have provided valuable insights into immediate responses to disturbance (Seidl and Turner 2022), they may not fully capture the complexity of post‐disturbance dynamics, which can take longer to stabilize (Adams 2001). Furthermore, because community‐level responses and turnover patterns often emerge after a time lag, evaluating mid‐ to long‐term trends facilitates distinguishing transient fluctuations from lasting shifts (Komatsu et al. 2019; Kaarlejärvi et al. 2021). Studies combining multi‐year post‐disturbance data with rigorous baseline data are crucial for accurately assessing recovery trajectories and predicting the future of ecological systems and their functions (Turner et al. 2003; Gonzalez et al. 2016; Gaiser et al. 2020). Despite the higher costs and logistical challenges, these mid‐ to long‐term studies offer deeper insights into ecosystem resilience and have greater potential to inform environmental policy and management (Hughes et al. 2017).

Insects are among the most threatened groups in the Anthropocene, with widespread declines raising concerns for biodiversity conservation and ecosystem stability (Dirzo et al. 2014; Harvey et al. 2023). As important contributors to ecosystem functions such as pollination, decomposition, and food web dynamics, shifts in insect communities can disrupt trophic interactions, alter recovery trajectories, and trigger cascading effects at the ecosystem level (Boyle et al. 2025). Their short generation times and high abundance also make them valuable indicators for assessing ecological responses to disturbances such as hurricanes, offering insights into resilience and recovery dynamics over relatively short timescales (Gerlach et al. 2013). Although insect responses to disturbance vary based on species‐specific tolerances, with both positive and negative outcomes reported in the literature (Schowalter 2012), increasingly severe disturbances may reduce overall species diversity as fewer species can withstand these extreme habitat or resource shifts (Haddad et al. 2008; Filazzola et al. 2021). Given the stochastic nature of hurricanes and the scarcity of long‐term datasets encompassing both pre‐ and post‐disturbance surveys, significant knowledge gaps exist regarding their impacts on insect taxa (Schowalter et al. 2017). This is especially true for moths, which play critical ecological roles yet remain underrepresented in long‐term disturbance studies, despite evidence of global biodiversity declines (Janzen and Hallwachs 2021; Wagner et al. 2021; San Blas and Devoto 2025).

In this study, we examined the temporal dynamics and ecological stability of tropical forest moth assemblages following large‐scale hurricane disturbance in Puerto Rico. While cyclonic storms are common disturbances in the Caribbean, Hurricane María in 2017 was the most intense storm to make landfall in nearly a century, with its extreme winds and rainfall drastically altering forested ecosystems island‐wide (Uriarte et al. 2019). We assessed changes in moth abundance, richness, and composition across two habitat types using monthly surveys conducted for 5 months before and 6 months after hurricane disturbance, followed by annual surveys for five consecutive years. To further explore the ecological stability of rainforest moth assemblages, we calculated resistance, resilience, recovery, and temporal stability for abundance, richness, and species composition using the framework developed by Hillebrand et al. (2018).

Previous post‐hurricane studies in the same forest have documented initial declines in Lepidoptera richness followed by full recovery within a year (Barberena‐Arias and Aide 2002), outbreaks in the abundance of species feeding on early successional vegetation (Torres 1992), and high species turnover through time (Schowalter et al. 2021). Based on these findings, we predicted: (1) decreased richness accompanied by abundance outbreaks during the first year of succession, driven by the initial loss of host‐specific and disturbance‐sensitive species and the rapid proliferation of grass‐feeding species; and (2) full recovery of abundance and richness within the study period, while species composition continues to shift in response to differences in dispersal ability, host plant availability, and the slower regeneration of forest structure and plant communities that support moth diversity. To the best of our knowledge, this six‐year dataset represents the longest available post‐hurricane record of moth assemblages, providing key insights into reassembly dynamics and the ecological stability of insect communities in a changing climate.

## 2 Materials and Methods

This study was conducted in the Luquillo Experimental Forest (LEF) within El Yunque National Forest, located in northeastern Puerto Rico. The LEF experiences a weakly seasonal climate, with a mean annual rainfall of 3500 mm and a mean monthly temperature of 23°C (González et al. 2014). The forest comprises both primary and secondary stands and serves as the site of the Luquillo Long‐Term Ecological Research (LUQ‐LTER) Program, which has monitored the effects of hurricane‐induced disturbances on tropical forests since the late 1980s (Zimmerman et al. 2021). In September 2017, the LEF was struck by two major hurricanes: Irma (category 5) and María (category 4). Hurricane María made direct landfall, resulting in extensive defoliation, high tree mortality, and significant changes to forest structure (Uriarte et al. 2019; Hall et al. 2020).

Moths were sampled in the lower montane forest near El Verde Field Station (18°20′ N, 65°49′ W; 200–600 m asl) on a monthly basis from April 2017 to March 2018 (excluding September 2017 due to hurricane disturbance) and annually during the summer months (July or August) from 2018 to 2022. Yearly summer sampling post‐hurricane was conducted during the same season as the pre‐hurricane monthly sampling to enable meaningful comparisons over time. We used automated light bucket traps (BioQuip Co., USA, model #2851) equipped with 22‐watt black lights, which ran throughout the night (19:00–05:00 h) in the understory (1–2 m above ground). Automated traps offer a standardized approach that enables simultaneous all‐night sampling across multiple locations (Brehm and Axmacher 2006), despite capturing only a subset of the moth community and slightly favoring larger species (Axmacher and Fiedler 2004; Merckx and Slade 2014).

Sampling was conducted at six sites: three in ridges dominated by *Dacryodes excelsa* (tabonuco; ~405 m asl) and three in valleys dominated by *Prestoea acuminata var. montana* (sierra palm; ~360 m asl), two of the most abundant tree species in this forest. Sites were separated by at least 80 m and sampled in random order during New Moon nights, with three sites visited per night, to minimize the confounding effects of moonlight on light‐trap catches (McGeachie 1989; Nowinszky et al. 2010). This design yielded six trap‐nights per sampling event (one per site) and ensured equal sampling effort across sites and months. While weather conditions can influence moth activity (Holyoak et al. 1997; Jonason et al. 2014), all traps ran simultaneously across sites during each sampling night, reducing bias from nightly variation. Canopy cover averaged 91% across both habitats before Hurricanes Irma and María, reflecting a mature and relatively uniform canopy structure (Appendix S1: Figure S1). The storms reduced cover to 24% and 17% in palm and tabonuco sites, respectively, but both had recovered to 95% by June 2020 and remained stable thereafter. Further details of our sampling methods and study sites are available in Alonso‐Rodríguez, Gutiérrez‐Fonseca, Agnarsson, et al. (2025).

Specimens with wingspans ≥ 1 cm were spread, sorted into morphospecies, and deposited at the University of Puerto Rico Museum of Entomology and Tropical Biodiversity (MEBT) in San Juan or the Smithsonian's National Museum of Natural History in Washington, DC. Preliminary species identifications were based on comparisons with museum collections and online reference materials. We sent leg tissue samples (Hebert et al. 2003) from at least one individual per morphospecies, usually more, to the Canadian Centre for DNA Barcoding for sequencing of the mitochondrial cytochrome *c* oxidase I (COI) gene. Finally, we verified species identifications through the Barcode of Life Data Systems (BOLD) platform, with support from expert taxonomists and comparisons with the Smithsonian Institution reference collection. The limited taxonomic knowledge of Puerto Rican moths (Terry et al. 2023) prevented identification of all morphospecies to the species level, so we used Barcode Index Numbers (BINs) as proxies for species identification where necessary (Ratnasingham and Hebert 2013).

### 2.1 Data Analysis

#### 2.1.1 Post‐Hurricane Temporal Dynamics

We used Linear Mixed‐effects Models (LMMs) to examine the effects of habitat, sampling month, and their interaction on moth abundance (total number of individuals) and species richness (total number of species). Models were based on a total of 96 site x night observations (48 per habitat type), with site included as a random effect to account for repeated sampling within locations, addressing potential non‐independence among observations. Models were fitted using the *lmer* function from the lme4 package (Bates et al. 2015), and the significance of fixed effects was evaluated using Analysis of Variance with the *anova* function from the stats package (R Core Team 2023). Model assumptions were verified by inspecting residuals for normality and homoscedasticity through diagnostic plots.

Model‐estimated means and confidence intervals for abundance and richness across habitat and month combinations were extracted using the *emmeans* function from the emmeans package (Lenth 2024). To evaluate temporal changes following the hurricanes, we performed post hoc contrast analyses by comparing estimated means of each post‐hurricane sampling month to the average abundance and richness observed during the pre‐hurricane sampling period, using the *contrast* function from the stats package (R Core Team 2023). The *p*‐values were adjusted using the Dunnett method (Dunnett 1955), which controls for multiple comparisons when testing several post‐hurricane time points against a single pre‐hurricane baseline.

We evaluated temporal dynamics in species composition using non‐metric multidimensional scaling (NMDS) based on Bray‐Curtis dissimilarities (Bray and Curtis 1957) of the site x night species abundance matrix. The NMDS was performed with two dimensions and 1000 random starts using the *metaMDS* function from the *vegan* package (Oksanen et al. 2022). To assess significant effects of habitat, sampling month, and their interaction on species composition, we applied a Two‐way Permutational Analysis of Variance (PERMANOVA). Pre‐hurricane months were grouped to establish a baseline for comparing post‐hurricane community composition. Lastly, we used contrast analyses to explore differences in species composition between each post‐hurricane sampling month and the baseline. To calculate these contrasts, individual PERMANOVA analyses were conducted, with permutations limited to include the baseline and the post‐hurricane month of interest, while controlling for the degrees of freedom from all other sampling months. The *p*‐values of these post hoc comparisons were adjusted with Holm's method (Holm 1979), which sequentially controls for multiple comparisons to reduce the risk of false positives while preserving statistical power. All PERMANOVAs were conducted using the *adonis2* function from the *vegan* package, with Bray‐Curtis distances and 1000 permutations. Analyses were conducted in R software version 4.3.2 (R Core Team 2023).

#### 2.1.2 Stability Analysis

Following the framework developed by Hillebrand et al. (2018), we calculated the four dimensions of stability (i.e., resistance, resilience, recovery, and temporal stability) for moth abundance, richness, and composition. Briefly, resistance quantifies the immediate deviation from the pre‐disturbance baseline, resilience captures the rate of change over time, recovery reflects the extent to which the system resembles the baseline by the end of the study period, and temporal stability captures the variability of these responses over time. Given that post‐hurricane responses can vary among taxa (Schowalter et al. 2017), we calculated these metrics for the entire moth community (all families combined) and separately for Crambidae, Erebidae, and Geometridae, three of the most abundant families in our collection.

Stability metrics for total abundance and richness, measured at the site x night level, were calculated using the same approach. Resistance was quantified as the log response ratio (LRR) between the first month after the hurricane (month 1) and a pre‐hurricane baseline, resilience as the slope of a regression of relative values (LRR) over time, recovery as the LRR between the final month of sampling (month 58) and a pre‐hurricane baseline, and temporal stability as the inverse of the standard deviation of residuals around the resilience trend (Hillebrand et al. 2018).

Compositional stability was assessed using the Bray–Curtis index (Bray and Curtis 1957), which quantified the abundance‐based similarity (1‐dissimilarity) between pre‐ and post‐hurricane communities. Compositional resistance was calculated as the similarity between the first month after the hurricane (month 1) and a pre‐hurricane baseline, resilience as the slope of a regression of similarity over time, recovery as the similarity between the final month of sampling (month 58) and a pre‐hurricane baseline, and temporal stability as the inverse of the standard deviation of residuals around the resilience trend (Hillebrand et al. 2018).

With five months of pre‐hurricane data available, we calculated resistance and recovery metrics five times per site, each using a different pre‐hurricane month as the baseline, and then averaged these values to obtain a single metric per site. This approach avoids any potential bias that could result from selecting a single month to represent pre‐hurricane conditions, which may vary due to natural temporal variation. By incorporating all available pre‐hurricane months, our estimates of resistance and recovery better reflect the typical baseline state of each site and provide more robust conclusions. This approach was not necessary for resilience and temporal stability, as their values remain unchanged regardless of which pre‐hurricane month is used as the baseline.

To test whether each stability metric differed significantly from expected values under undisturbed conditions, we conducted One‐Sample t‐Tests against their corresponding pre‐hurricane benchmarks (Hillebrand et al. 2018; Capdevila et al. 2021). Unlike the baselines mentioned above, which refer to the individual pre‐hurricane months used to calculate the metrics, benchmarks represent expected stability values derived from the natural variability among those months. We developed benchmarks for resistance and recovery by averaging all pairwise comparisons between pre‐hurricane months (using LRR for abundance and richness, and Bray–Curtis similarities for composition) for each site, and then averaging across all sites to obtain an overall benchmark for each family. For resilience, we used 0 as the benchmark, representing no directional change over time (i.e., a flat slope). We did not run t‐Tests for temporal stability as it has no benchmark; instead, higher temporal stability indicates lower fluctuations around the resilience trend. Although metrics were calculated separately for each site, we pooled data across habitats for the t‐Tests to streamline the analysis and highlight broad patterns of community and family‐level stability across the lower montane forest. Separate habitat‐level analyses would have doubled the number of comparisons, reduced replication, and significantly complicated interpretation. One‐Sample t‐Tests were run with the *t.test* function from the stats package in R (R Core Team 2023).

## 3 Results

Over the course of 16 sampling events spanning 6 years at six sites within the Luquillo Experimental Forest (LEF), we collected a total of 9635 moths. From these, 9462 individuals were assigned to 318 morphospecies, with 295 classified to genus and 206 to species. Of all morphospecies, 24% were assigned to Barcode Index Numbers (BINs) but could not be identified to species level. A small fraction of individuals (1.8%) was too damaged to identify. The collected moths were distributed across 21 families, with the greatest species richness in Crambidae (27.4%), Erebidae (20.8%), Pyralidae (11.6%), and Geometridae (11.3%). Although there is no modern taxonomically validated list of the moths of Puerto Rico, Torres and Medina Gaud (1998) reported 1045 species of Lepidoptera, of which our study captured nearly one‐third (~30%) within a single forest system. Rarefaction and extrapolation analyses further indicated asymptotic species richness of 317 species in the palm forest (255 observed, 80% of estimated richness) and 288 species in the tabonuco forest (261 observed, 91% of estimated richness), suggesting relatively high sampling completeness (Appendix S1: Figure S2). A notable portion of the species pool consisted of rare taxa, with 28% represented by a single individual (singletons) and 12% by two individuals (doubletons), which is typical when sampling tropical insects (Novotný and Basset 2000). Of the 318 morphospecies recorded, 42 were detected only before the passage of Hurricanes Irma and María, and 144 were detected only after the hurricanes. A complete list of species, along with their total counts in palm and tabonuco stands, is provided in Appendix S2: Table S1.

### 3.1 Shifts in Moth Communities Across Time and Habitats

**FIGURE 1:**
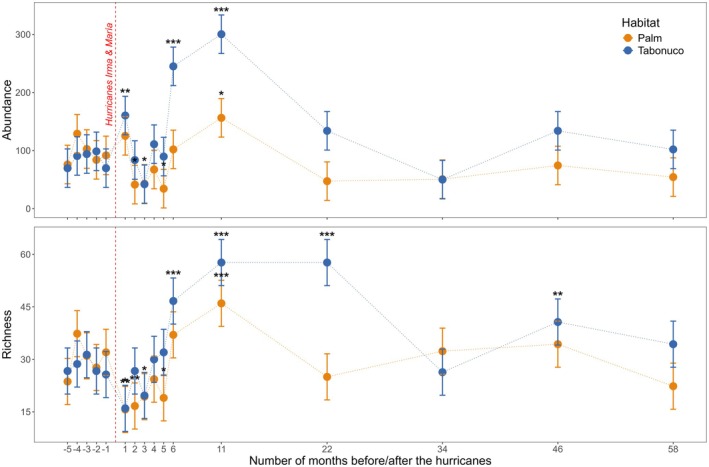
Abundance and richness dynamics of moth communities in palm and tabonuco stands. Points represent the estimated marginal means ±95% confidence intervals from Linear Mixed‐effects Models (LMMs). Sampling months are shown on the x‐axis, with negative values representing pre‐hurricane months and positive values representing post‐hurricane months, where month 0 corresponds to September 2017. Months that differ significantly from the pre‐hurricane average are denoted by asterisks, with *** indicating highly significant differences (*p* < 0.001), ** for moderately significant differences (*p* < 0.01), and * for slightly significant differences (*p* < 0.05).

Moth abundance varied significantly over time and between habitats, with distinct temporal patterns observed in each habitat (LMM; Table 1). Nightly abundance was similar between habitats before the hurricanes, with substantial overlap in the confidence intervals across pre‐hurricane months indicating limited baseline variability (Figure 1). After the hurricanes, abundance patterns diverged, with tabonuco generally supporting higher abundance than palm forests. Abundance in tabonuco stands surged 1 month post‐hurricane, more than doubling the pre‐hurricane average, and peaked at 6 and 11 months, reaching over 4 times the pre‐hurricane average. In contrast, abundance in palm stands dropped to less than half of the pre‐hurricane average 2 months post‐hurricane and remained low through month 5, after which it started to increase, peaking at month 11 with twice the pre‐hurricane average. By month 22 (almost 2 years after the hurricanes), abundance in both habitats had returned to pre‐hurricane levels and remained stable over the following 3 years (Figure 1).

**TABLE 1:** Results of Linear Mixed‐effects Models (LMMs) and two‐way PERMANOVA to evaluate the effects of sampling month, habitat (palm vs. tabonuco), and their interaction on abundance, richness, and composition of moth communities. Site was included as a random effect in LMMs, while pre‐hurricane months were grouped in the PERMANOVA.

	Sum Sq	Mean Sq	*R* ^2^	NumDF	DenDF	*F*‐value	*p*
Abundance (LMM)							
Month	205,804	13,720	—	15	60	16.61	**< 0.0001**
Habitat	32,857	32,857	—	1	4	39.78	**0.0032**
Month*Habitat	63,971	4265	—	15	60	5.16	**< 0.0001**
Richness (LMM)							
Month	7528.5	501.9	—	15	60	15.99	**< 0.0001**
Habitat	401.0	401.0	—	1	4	12.78	**0.0233**
Month*Habitat	2269.3	151.3	—	15	60	4.82	**< 0.0001**
Composition (PERMANOVA)							
Month	13.1	—	0.49	11	72	8.03	**0.0010**
Habitat	0.6	—	0.02	1	72	4.17	**0.0010**
Month*Habitat	2.3	—	0.08	11	72	1.38	**0.0030**

*Note:* Significant effects at α = 0.05 are highlighted in bold.

Similar to abundance, species richness exhibited distinct temporal patterns between habitats (LMM; Table 1), with comparable values in both habitats before the hurricanes but diverging post‐hurricane (Figure 1). In tabonuco stands, richness declined immediately after the hurricanes but quickly rebounded, reaching 1.5 times the pre‐hurricane average by month 6 and staying elevated for nearly 2 years, peaking at more than twice the pre‐hurricane average by month 22. In contrast, richness in palm stands dropped significantly post‐hurricane (month 1), remained low until month 5, and then increased, peaking at month 11 with 1.5 times the pre‐hurricane average. By month 22, richness in palm stands had returned to pre‐hurricane levels and remained stable through the final sampling event. However, richness in tabonuco stands continued to fluctuate, with another significant surge at month 46 (Figure 1).

**FIGURE 2:**
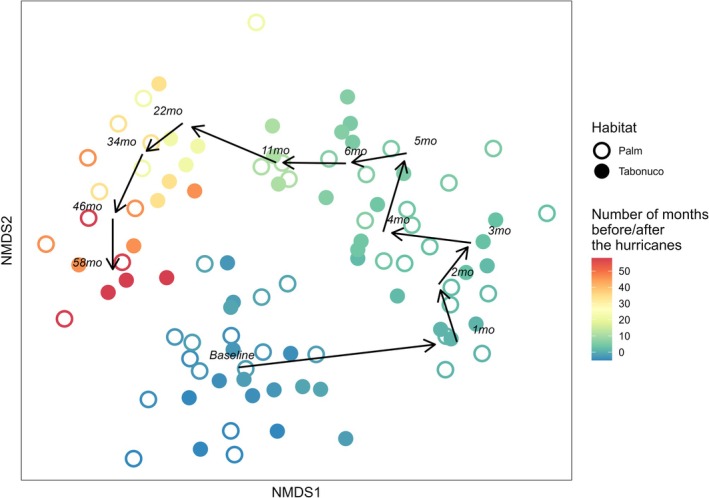
NMDS ordination of species composition over time in palm and tabonuco stands (stress = 0.26). The color gradient represents sampling months, with bluer shades indicating pre‐hurricane months (months −5 to −1) and transitioning from green to red for post‐hurricane months (months 1 to 58), with month 0 representing September 2017. Black arrows depict the temporal progression of centroids for each post‐hurricane sampling event. Habitat types were pooled for the centroid calculations to emphasize broader temporal dynamics.

Species composition underwent continuous shifts over the five years following the hurricanes, with communities in both habitats changing at each sampling event. As shown in the NMDS ordination (Figure 2), there was a noticeable shift in composition immediately after the hurricanes (month 1), with communities diverging from the pre‐hurricane baseline. Communities continued to occupy distinct positions in ordination space across time points, reflecting sustained species turnover throughout the study period. Temporal patterns varied between palm and tabonuco stands, as evidenced by a significant interactive effect of month and habitat on species composition (two‐way PERMANOVA; Table 1). Despite the apparent trajectory toward recovery, composition in all post‐hurricane months (months 1 through 58) remained significantly different from the pre‐hurricane baseline (*p* < 0.05 for all post hoc contrasts per habitat).

### 3.2 Ecological Stability of Moth Communities

**FIGURE 3:**
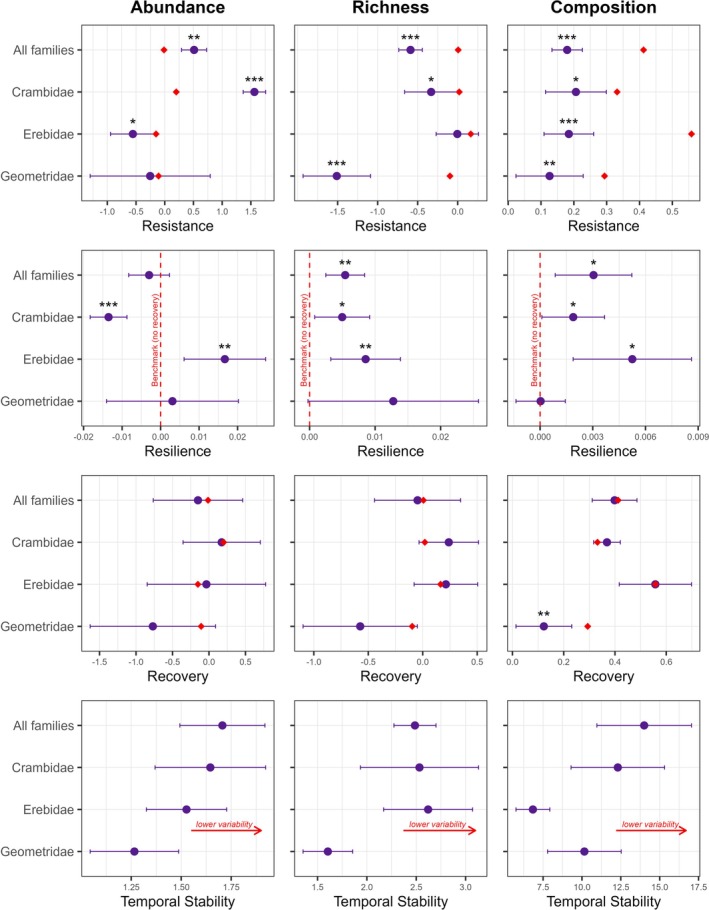
Stability metrics for moth abundance (left column), richness (middle column), and composition (right column). We calculated resistance, resilience, recovery, and temporal stability for all families combined and separately for Crambidae, Erebidae, and Geometridae. Metrics are based on log response ratios for abundance and richness and Bray‐Curtis similarities for composition. Red triangles in resistance and recovery plots represent benchmarks for each family, while benchmarks in resilience plots are marked with red dashed vertical lines at 0. Metrics that differ significantly from their pre‐hurricane benchmarks are denoted by asterisks, with *** indicating highly significant differences (*p* < 0.001), ** for moderately significant differences (*p* < 0.01), and * for slightly significant differences (*p* < 0.05). Resistance and recovery metrics indicate how much abundance, richness, and composition deviated from pre‐hurricane benchmarks in the first and last month of sampling post‐hurricane, respectively. For abundance and richness, values above the benchmark reflect an increase, while values below reflect a decrease. Composition values range from 0 to 1, with 1 representing higher similarity to pre‐hurricane conditions. Resilience represents the rate of change, with positive or negative values representing the directionality of the response over time. Temporal stability captures the variability in each metric during recovery, with lower values indicating greater fluctuation. Refer to Appendix S1: Table S1 for full results of one‐sample *t*‐tests and additional details on metric interpretation.

Using the time series data presented in Figures 1 and 2, we estimated and analyzed the ecological stability metrics introduced by Hillebrand et al. (2018). Moth assemblages exhibited low resistance to hurricane disturbance across abundance, richness, and composition (Figure 3, top row). Immediately following the hurricanes, total moth abundance increased significantly relative to the pre‐hurricane benchmarks, whereas richness and compositional similarity decreased significantly (Appendix S1: Table S1). A similar response was observed for Crambidae, the most abundant family in our collection. Erebidae also exhibited significant shifts in both abundance and composition, though richness remained unchanged. In contrast, Geometridae experienced a sharp decline in richness and shifts in composition, while abundance was unaffected, likely due to high site‐to‐site variation (Figure 3).

Resilience in richness and composition was significantly positive across all families as well as for Crambidae and Erebidae (resilience > 0), indicating a gradual return toward pre‐hurricane benchmarks (Figure 3, second row). Meanwhile, patterns in abundance varied. Erebidae abundance exhibited a positive resilience slope, whereas Crambidae showed a negative resilience slope, reflecting trends returning to benchmark from opposite directions (Appendix S1: Table S1). The resilience in abundance for all families combined was low, as indicated by flat slopes (resilience not different from 0). In contrast to other families, Geometridae showed low resilience overall (Figure 3).

Five years after the hurricanes, moth assemblages had returned to their pre‐hurricane benchmarks in abundance, richness, and composition (Figure 3, third row). This recovery was consistent across all families assessed, except for Geometrid composition, which remained distinct from the benchmark (Appendix S1: Table S1). Temporal stability was also similar across families, except for Geometridae, which exhibited higher temporal variability in abundance and richness (Figure 3, bottom row). While Geometridae also showed low temporal stability in composition, Erebidae exhibited even lower stability, demonstrating the highest compositional variability over time.

## 4 Discussion

By monitoring tropical forest moth communities for 5 years after two extreme hurricanes, this study offers critical insights into their dynamic responses and recovery patterns following large‐scale disturbance. As predicted, moth abundance and species richness rebounded within 2 years after Hurricanes Irma and María in Puerto Rico, despite short‐term fluctuations during early succession. Meanwhile, species composition showed a directional trajectory toward recovery, but full compositional recovery was not definitively confirmed even 5 years post‐disturbance, indicating ongoing species turnover. Responses varied by moth family and habitat type, with the forest‐dependent Geometridae exhibiting greater long‐term compositional changes, while tabonuco stands supported moth communities more effectively than palm stands during early succession. Overall, our results point to relatively high functional and compositional stability in these assemblages, though direct measurements of function and continued monitoring of composition will be important to corroborate these patterns. The key challenge will be determining whether this resilience can persist under increasingly frequent and intense disturbance regimes driven by global climate change.

### 4.1 Post‐Hurricane Response Trajectories

Disturbance often triggers spikes in insect abundance, typically driven by outbreaks of herbivorous or disturbance‐tolerant taxa that can quickly exploit altered habitats (Schowalter 2012; Novais et al. 2018). In contrast, species richness generally declines post‐disturbance (Summerville and Crist 2002; Murphy and Romanuk 2014; Mangels et al. 2017; Banza et al. 2019), as seen in our earlier analysis covering the first 6 months after Hurricanes Irma and María (Alonso‐Rodríguez, Gutiérrez‐Fonseca, Agnarsson, et al. 2025). In this study, we demonstrate an overall decline in moth abundance and richness during this period. Similar patterns in insect communities have been linked to increased insect mortality due to high winds and water flow, as well as resource scarcity and altered abiotic conditions following widespread defoliation (White and Jentsch 2001; Koptur et al. 2002; Schowalter 2012).

This decline was temporary, as both abundance and richness surged 6 months post‐hurricane, peaking within the first year at levels exceeding pre‐hurricane baselines. While defoliation and structural damage from the hurricanes may have increased light trap visibility and slightly inflated captures, studies show that weak light traps attract moths over very short distances (often less than 10 m) and primarily sample the local community (Truxa and Fiedler 2012). This suggests that the observed post‐hurricane surge likely reflects genuine local population increases rather than movement from distant areas. Additionally, our findings align with Torres (1992), who documented outbreaks of 15 Lepidoptera species feeding on young, early successional foliage in the Luquillo Mountains ~7 months after Hurricane Hugo in 1989. Meanwhile, the surge in richness may reflect species colonization from surrounding areas, as the open post‐hurricane landscape likely promotes greater species dispersal in search of suitable habitat and resources (Vanschoenwinkel et al. 2013; Meyer et al. 2021).

Abundance and richness spikes in the first year post‐hurricane were more pronounced in tabonuco forest stands compared to palm stands, likely reflecting differences in forest structure and microclimatic conditions during succession (La Cava et al. 2024). Palm stands, which occur primarily in riparian valleys, tend to have shorter, simpler canopies and often lose most of their fronds during storms (Walker 1991; Lugo and Frangi 2016; Uriarte et al. 2023). This can lead to higher understory temperatures and harsher conditions for rainforest insects (Harvey et al. 2020). In contrast, tabonuco stands are typically found on ridges and maintain taller, more complex canopies that may help sustain moth communities by providing a wider range of microhabitats, including sheltered spaces where larvae and pupae can persist (Lugo and Wadsworth 1990; Waide 1991; Weaver 2010). In addition, canopy cover in tabonuco stands showed slightly faster recovery in the first year post‐hurricane compared to palm stands (Appendix S1: Figure S1). Such structural recovery may create conditions similar to disturbance refugia, providing shelter and critical resources for animal communities during early succession (Krawchuk et al. 2020; Vangansbeke et al. 2025). This highlights the conservation value of old‐growth tabonuco forests, which once dominated Puerto Rico's central mountains and lowlands but were largely lost to agricultural expansion in the early 20th century (Álvarez Ruiz and Lugo 2012).

Applying the Hillebrand stability framework (Hillebrand et al. 2018) confirmed that resistance to hurricanes varies across moth families (Figure 3), which is consistent with prior research (Schowalter 2012; Filazzola et al. 2021). Crambidae exhibited the greatest increase in abundance during the first month post‐hurricane (Appendix S1: Table S1). Their small size may contribute to shorter generation times and reduced predation risk (Dillon and Frazier 2013; Rabl et al. 2020), while their stem‐boring feeding strategy may have provided protection from wind and debris (Rathikannu and Chitra 2017). In addition, crambid larvae primarily feed on grasses (Henaish and El‐Metwaly 2023), which dominate in the forest during the early stages of post‐hurricane succession (Lugo 2008). Conversely, Geometridae experienced the most pronounced species loss immediately after the hurricanes (Figure 3), aligning with previous studies that identify geometrids as particularly vulnerable to habitat disturbance (Intachat et al. 2001; Hilt et al. 2006; Alonso‐Rodríguez et al. 2017).

Despite low overall resistance to hurricanes, our data indicate that moth abundance and richness recovered fully within 2–3 years (Figure 1), with all three families returning to benchmark levels by our last sampling event, 5 years post‐hurricane (Figure 3). This contrasts with Barberena‐Arias and Aide (2002), who reported full recovery of Lepidoptera communities only a year after Hurricane Georges in 1998. The longer recovery time observed in our study may reflect the greater severity of Hurricane María, which resulted in higher tree mortality and structural damage than any other hurricane in recent decades (Uriarte et al. 2019). However, considering the intensity of María, the recovery observed in our study can still be considered relatively swift and may be driven in part by stabilizing mechanisms within the trophic cascade (Chesson 2000). For instance, Torres (1992) documented that post‐hurricane moth outbreaks in Puerto Rico were regulated by Dipteran and Hymenopteran parasitoids, while Spiller et al. (2016) found that *Anolis* lizards played a key role in controlling moth populations in the Bahamas, where islands with lizards had significantly lower moth abundance and greater plant recovery following hurricane disturbance. Given that anole lizards are also key predators of Lepidoptera at our study site (Terry et al. 2023), their presence may similarly contribute to regulating moth outbreaks and facilitating vegetation recovery.

Research has shown that multivariate assessments of composition often reveal lasting changes that univariate diversity measures fail to capture (Stork et al. 2017). Our findings support this, as community composition exhibited longer‐lasting impacts of disturbance than abundance and richness, remaining distinct from baseline even 5 years post‐hurricane (Figure 2). Disturbance effects on composition can persist due to many factors, including barriers to re‐colonization, shifts in species interactions, or the dynamic and ongoing changes in abiotic and biotic conditions during succession (Barberena‐Arias and Aide 2002; Hillebrand and Kunze 2020). Additionally, priority effects and environmental filtering can determine community reassembly after disturbance (Fukami 2015), leading to persistent shifts in composition over time, as observed in this study. The challenges for compositional recovery may be even greater on islands due to their geographic isolation, which limits access to external species pools and hinders recolonization (Gillespie et al. 2008).

We found an immediate shift in species composition across both habitat types following the hurricanes (Figure 2). This was supported by our stability metrics, which showed low compositional resistance for all families combined, as well as for each of the three individual families assessed (Figure 3). However, these metrics also indicated full recovery by the last sampling event (58 months post‐hurricane; Appendix S1: Table S1), in contrast with results from NMDS ordination and PERMANOVA (Table 1, Figure 2). This apparent discrepancy likely reflects fundamental differences in how each approach measures compositional change. PERMANOVA tests for statistical differences in the multivariate centroids of community composition (Anderson 2017), capturing directional changes in species identity and abundance patterns over time. Consistent with this, our NMDS ordination shows a directional trajectory toward the baseline but not a full return (Figure 2), suggesting that the community is still undergoing reassembly. In contrast, we calculated compositional recovery as the average Bray–Curtis similarity between post‐hurricane communities and multiple pre‐hurricane baselines, contextualizing recovery relative to natural background variation (Hillebrand et al. 2018). Since Bray‐Curtis is sensitive to the presence and relative abundance of shared species, it can yield high similarity values even when communities differ in species dominance or structure (Ricotta and Podani 2017). As such, this metric may indicate recovery has occurred even if the community has not returned to its original multivariate configuration. Together, these findings underscore how methodological differences can lead to contrasting interpretations and highlight the importance of using complementary approaches to assess long‐term ecological change.

### 4.2 Implications for the Compositional and Functional Stability of Moth Communities

Evaluating the relationship between biodiversity and ecosystem function has long been a central focus in ecology, particularly in the context of disturbance and environmental change (Loreau et al. 2001; Hooper et al. 2005). Philippot et al. (2021) proposed that full recovery occurs when both species composition and function return to pre‐disturbance conditions, whereas functional redundancy allows function to persist despite compositional shifts, and physiological adaptation occurs when species composition recovers but function does not. Using the Hillebrand et al. (2018) stability framework, we assessed compositional recovery and can infer functional recovery based on moth abundance (Figure 3). While debate remains on whether species richness, specific taxa, or overall abundance best predict ecosystem function (Winfree 2020), several studies have shown that abundance or biomass can serve as a useful proxy for evaluating changes in ecological processes (Davies et al. 2011; Gaston et al. 2018; Sundstrom et al. 2018; Hillebrand and Kunze 2020). For instance, Winfree et al. (2015) demonstrated that in crop pollination systems, fluctuations in the abundance of dominant species were more important for ecosystem service delivery than changes in species richness. By analogy, the complete recovery of moth abundance in our study suggests that key trophic interactions, such as their roles in food webs and plant‐pollinator networks, may have been maintained despite ongoing changes in richness or species composition.

At the community level, our stability metrics indicate full recovery of both compositional and functional aspects of moth assemblages 5 years after Hurricanes Irma and María (Figure 3). This aligns with previous research showing that the Puerto Rican biota is well adapted to disturbance and capable of relatively rapid recovery following large‐scale events (Zimmerman et al. 2020; Schowalter et al. 2021; Presley and Willig 2023). However, the extent of compositional recovery should be interpreted with caution, as PERMANOVA results suggest continued community reassembly rather than a complete return to pre‐hurricane composition. This may indicate that functional redundancy plays a key role in this system, buffering the effects of disturbance as some species compensate for the loss or decline of others and maintain essential ecological functions despite shifts in species identity (Gonzalez and Loreau 2009; Philippot et al. 2021).

Previous studies on insect responses to hurricane disturbance in this system have found that shifts in biodiversity and abundance are largely driven by changes in forest structure and composition during succession (Schowalter 2017; Schowalter et al. 2017). Our study supports these findings and highlights the importance of long‐term data in capturing the temporal dynamics of recovery and their implications for ecosystem functioning (Turner et al. 2003). In a study of post‐hurricane salt‐marsh insect communities along the Mississippi River in Louisiana, Chen et al. (2020) proposed the low resistance/high resilience hypothesis to explain how insect communities recovered within just one year following category 1 Hurricane Isaac, despite initial declines in diversity and shifts in composition. While differences in ecosystem type, hurricane intensity, and focal taxa must be considered, a similar framework may help explain the post‐hurricane recovery patterns of both composition and function observed in our study. The ability of the moth community to recover within 5 years, despite the unprecedented strength of Hurricane María and ongoing forest succession, underscores the resilience of tropical insect communities in hurricane‐prone forests.

To better understand functional stability, future research should integrate trait‐based approaches or direct measurements to assess whether species replacements lead to shifts in ecosystem function. Unfortunately, such analyses remain constrained by the limited available data on key functional traits, such as feeding guilds, dispersal ability, and life‐history strategies (Terry et al. 2023). Additionally, studies incorporating higher‐resolution temporal data (e.g., sampling across different seasons) and experimental manipulations could clarify the mechanisms underlying the resilience of moth communities, shedding light on whether functional and compositional stability persist under novel disturbance regimes. Given the increasing frequency of large‐scale hurricanes due to climate change, advancing our understanding of resilience thresholds and the potential for long‐term compositional and functional shifts will be critical for predicting the future stability of tropical biodiversity.

## Author Contributions

**Aura M. Alonso‐Rodríguez:** conceptualization (lead), data curation (lead), formal analysis (lead), funding acquisition (supporting), investigation (lead), methodology (lead), project administration (lead), supervision (lead), visualization (lead), writing – original draft (lead), writing – review and editing (lead). **Pablo E. Gutiérrez‐Fonseca:** conceptualization (lead), formal analysis (supporting), investigation (supporting), methodology (supporting), visualization (supporting), writing – review and editing (supporting). **Scott E. Miller:** data curation (supporting), funding acquisition (supporting), resources (supporting), writing – review and editing (supporting). **Taylor H. Ricketts:** formal analysis (supporting), funding acquisition (supporting), validation (supporting), writing – review and editing (supporting).

## Conflicts of Interest

The authors declare no conflicts of interest.

## Supporting information


**Appendix S1:** ece372278‐sup‐0001‐Supinfo01.pdf.

**Appendix S2:** ece372278‐sup‐0002‐Supinfo02.pdf.

## Data Availability

The code and data supporting this study (Alonso‐Rodríguez, Gutiérrez‐Fonseca, Miller, et al. 2025) are publicly available on Figshare at https://doi.org/10.6084/m9.figshare.29405807.v1.
