# Supplementary material for: Moth Communities Reveal High Stability Despite Ongoing Compositional Shifts Over Five Years Following Hurricane Disturbance
Source: Ecol Evol. 2025 Oct 9;15(10):e72278. doi: 10.1002/ece3.72278 (PMC12511569; doi:10.1002/ece3.72278)
Supplement: Supplementary file 1 — Appendix S1: ece372278‐sup‐0001‐Supinfo01.pdf. [file ECE3-15-e72278-s001.pdf]

Supporting Information. Aura M. Alonso-Rodríguez, Pablo E. Gutiérrez-Fonseca, Scott E. Miller, and Taylor H. Ricketts. Moth communities reveal high stability despite ongoing compositional shifts over five years following hurricane disturbance.

Appendix S1. Supplementary figures and tables

Figure S1. Mean canopy cover (%) ± standard errors in palm and tabonuco stands throughout the study period. Red vertical lines indicate the timing of Hurricanes Irma and María. Canopy cover was measured at each sampling site using a spherical convex densiometer on the following dates: 6-Sep-2017 (right before Hurricane Irma), 10-Sep-2017 (after Irma), 4-Oct-2017 (first measurement after Hurricane María), 30-Nov-2017, 25-Jan-2018, 28-Feb-2018, 3-Apr-2018, 15-Aug-2018, 8-Aug-2019, 20-Jun-2020, 27-Jul-2021, and 3-Aug-2022.

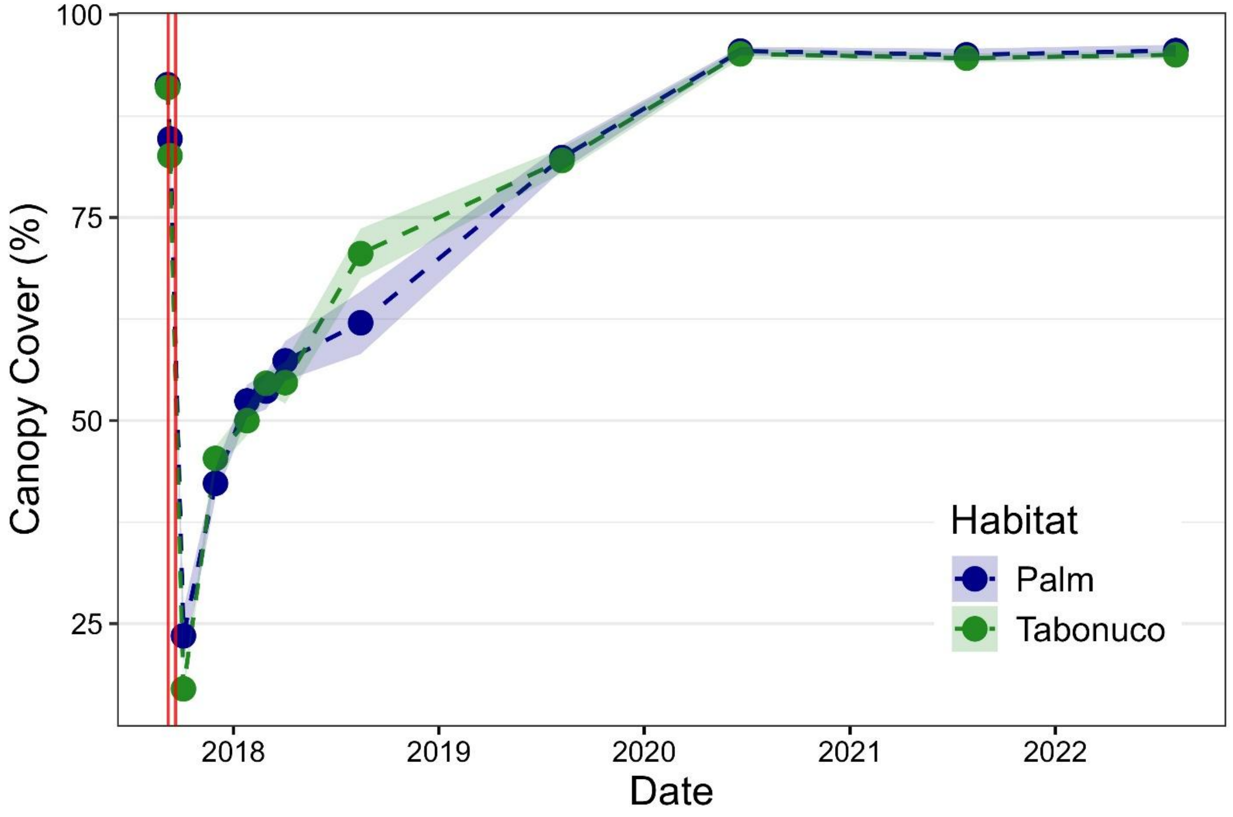

Figure S2. To evaluate sampling completeness and estimate total richness in palm and tabonuco forests, we used the iNEXT.3D Online platform (Chao and Hu 2024) to generate rarefaction and extrapolation curves for species richness (Hill number q = 0), following the framework of Chao et al. (2021). The sample-size–based (top) and coverage-based (bottom) curves show observed richness of 255 species in palm and 261 species in tabonuco (symbols), with asymptotic richness estimated at 317 species for palm and 288 species for tabonuco. These estimates indicate relatively high sampling completeness, capturing ~80% of the estimated species pool in palm and ~91% in tabonuco. Solid lines represent rarefaction, dashed lines extrapolation, and shaded areas show the 95% confidence intervals, calculated with 1,000 bootstrap replicates and 40 equally spaced knots. Overall, these results suggest that palm stands may harbor slightly higher species richness than tabonuco stands.

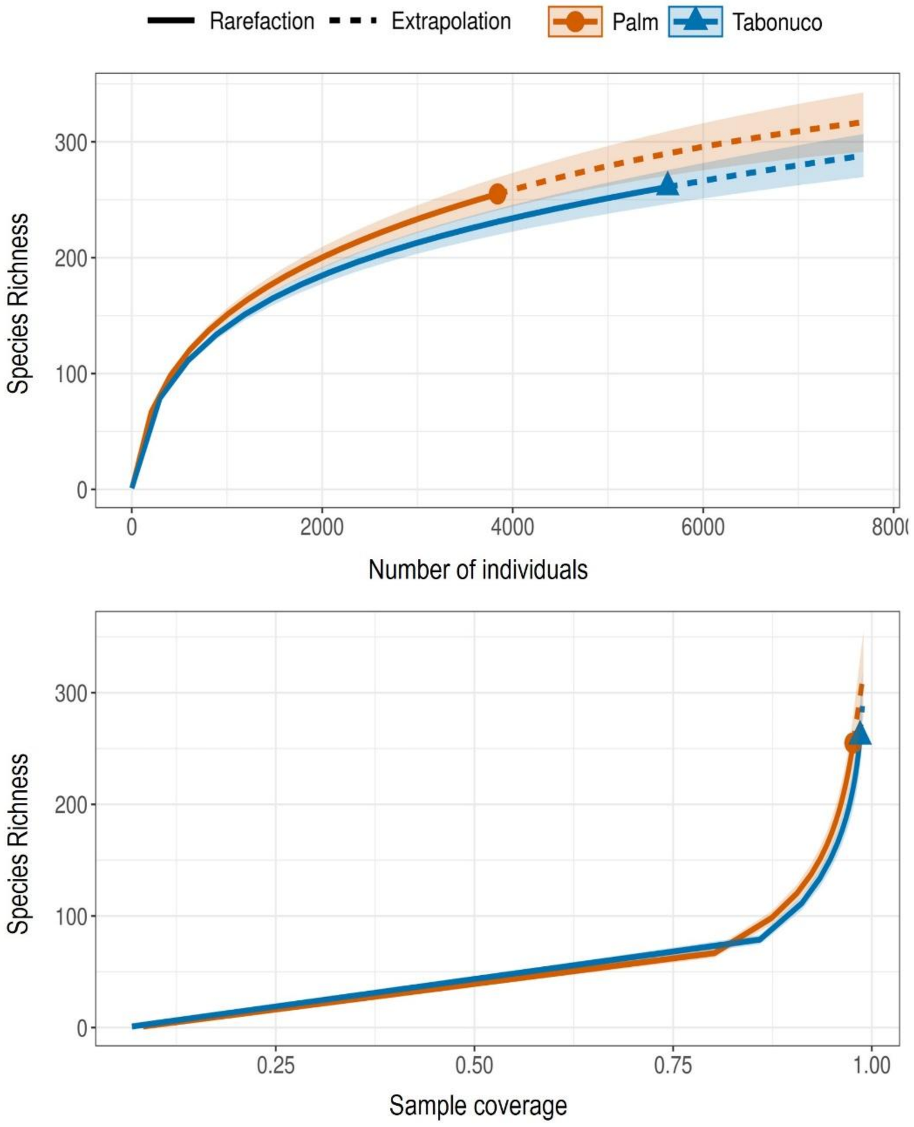

Table S1. Results of one-sample t-tests comparing post-hurricane resistance, recovery, and resilience of moth assemblages in the Luquillo Experimental Forest, Puerto Rico, against benchmark values. Analyses were conducted for all families combined and separately for Crambidae, Erebidae, and Geometridae. Although metrics were calculated separately by site, data were pooled across habitats for the t-tests (df = 5) to emphasize broader patterns. We report t-values, p-values, means, and 95% confidence intervals for each metric, along with the benchmark values used for comparison. Benchmarks for resistance and recovery were obtained by averaging all pairwise comparisons between pre-hurricane months (using log response ratios for abundance and richness and Bray–Curtis similarity for composition) per site, and then averaging across sites. Resilience was assessed using a benchmark of 0, representing no recovery (a flat slope over time). Temporal stability was not tested due to the absence of a benchmark. We include an interpretation column to clarify how to interpret significant p-values.

	All families	Crambidae	Erebidae	Geometridae	Interpretation
Abundance					
Resistance					
T-value	6.156	17.904	-2.657	-0.360	p<0.05 indicates *low resistance* (i.e., altered species abundances 1mo. post-hurricane)
p-value	**0.00165**	**0.00001**	**0.04503**	0.73370	
Mean	0.511	1.562	-0.555	-0.254	
95% CI	[0.292, 0.730]	[1.366, 1.757]	[-0.944, -0.165]	[-1.299, 0.792]	
Benchmark	-0.013	0.201	-0.152	-0.107	
Recovery					
T-value	-0.579	-0.130	0.369	-2.139	p<0.05 indicates *low recovery* (i.e., abundance had not returned to baseline 58mo. post-hurricane)
p-value	0.58790	0.90190	0.72750	0.09917	
Mean	-0.151	0.174	-0.035	-0.769	
95% CI	[-0.764, 0.462]	[-0.355, 0.703]	[-0.847, 0.776]	[-1.629, 0.090]	
Benchmark	-0.013	0.201	-0.152	-0.107	
Resilience					
T-value	-1.446	-7.264	4.049	0.462	p<0.05 indicates *high resilience* (i.e., rapid rate of recovery of species abundances)
p-value	0.20790	**0.00077**	**0.00983**	0.66330	
Mean	-0.003	-0.014	0.017	0.003	
95% CI	[-0.008, 0.002]	[-0.018, -0.009]	[0.006, 0.027]	[-0.014, 0.020]	
Benchmark	0	0	0	0	
Richness					
Resistance					
T-value	-10.429	-2.723	-1.631	-8.625	p<0.05 indicates *low resistance* (i.e., altered species richness 1mo. post-hurricane)
p-value	**0.00014**	**0.04163**	0.1638	**0.00035**	
Mean	-0.589	-0.331	-0.004	-1.511	
95% CI	[-0.736, -0.442]	[-0.663, -0.000]	[-0.270, 0.261]	[-1.932, -1.089]	
Benchmark	0.006	0.020	0.164	-0.097	

Recovery					
T-value	-0.350	2.068	0.429	-2.526	p<0.05 indicates *low recovery* (i.e., richness had not returned to baseline 58mo. post-hurricane)
p-value	0.74090	0.09343	0.68580	0.06493	
Mean	-0.047	0.239	0.212	-0.574	
95% CI	[-0.443, 0.348]	[-0.034, 0.513]	[-0.079, 0.504]	[-1.099, -0.049]	
Benchmark	0.006	0.020	0.164	-0.097	
Resilience					
T-value	4.711	3.050	4.137	2.5211	p<0.05 indicates *high resilience* (i.e., rapid rate of recovery of species richness)
p-value	**0.00529**	**0.02842**	**0.00903**	0.05311	
Mean	0.005	0.005	0.009	0.013	
95% CI	[0.002, 0.008]	[0.001, 0.009]	[0.003, 0.014]	[-0.000, 0.026]	
Benchmark	0	0	0	0	
Composition					
Resistance					
T-value	-12.906	-3.480	-12.701	-4.204	p<0.05 indicates *low resistance* (i.e., altered species composition 1mo. post-hurricane)
p-value	**0.00005**	**0.01765**	**0.00005**	**0.00846**	
Mean	0.180	0.206	0.185	0.126	
95% CI	[0.133, 0.226]	[0.114, 0.299]	[0.109, 0.260]	[0.024, 0.229]	
Benchmark	0.412	0.332	0.558	0.294	
Recovery					
T-value	-0.403	1.836	-0.013	-4.043	p<0.05 indicates *low recovery* (i.e., composition had not returned to baseline 58mo. post-hurricane)
p-value	0.70340	0.12570	0.99040	**0.00990**	
Mean	0.399	0.369	0.557	0.123	
95% CI	[0.311, 0.486]	[0.317, 0.421]	[0.417, 0.698]	[0.014, 0.231]	
Benchmark	0.412	0.332	0.558	0.294	
Resilience					
T-value	3.585	2.716	4.006	0.057	p<0.05 indicates *high resilience* (i.e., rapid rate of recovery of species composition)
p-value	**0.01579**	**0.04196**	**0.01026**	0.95650	
Mean	0.003	0.002	0.005	0.000	
95% CI	[0.001, 0.005]	[0.000, 0.004]	[0.002, 0.009]	[-0.001, 0.001]	
Benchmark	0	0	0	0	

References

Chao, A., Henderson, P. A., Chiu, C.-H., Moyes, F., Hu, K.-H., Dornelas, M. and Magurran, A. E. 2021. Measuring temporal change in alpha diversity: a framework integrating taxonomic, phylogenetic and functional diversity and the iNEXT.3D standardization. Methods in Ecology and Evolution 12: 1926-1940.

Chao, A. and Hu, K.-H. 2024. iNEXT.3D Online: interpolation and extrapolation for three dimensions of biodiversity. Code and user's guide available from https://chao.shinyapps.io/iNEXT_3D
