# Supplementary material for: Moth Communities Reveal High Stability Despite Ongoing Compositional Shifts Over Five Years Following Hurricane Disturbance
Source: Ecol Evol. 2025 Oct 9;15(10):e72278. doi: 10.1002/ece3.72278 (PMC12511569; doi:10.1002/ece3.72278)
Supplement: Supplementary file 2 — Appendix S2: ece372278‐sup‐0002‐Supinfo02.pdf. [file ECE3-15-e72278-s002.pdf]

Supporting Information. Aura M. Alonso-Rodríguez, Pablo E. Gutiérrez-Fonseca, Scott E. Miller, and Taylor H. Ricketts. Moth communities reveal high stability despite ongoing compositional shifts over five years following hurricane disturbance.

Appendix S2. Inventory of moth species detected throughout the study period

Table S1. Full list of moth taxa and their recorded abundances from Sierra Palm (P) and Tabonuco (T) stands in the Luquillo Experimental Forest, Puerto Rico, collected between April 2017 and July 2022. Species codes correspond to project-assigned identifiers for each morphospecies. Codes marked with (–) indicate taxa detected only before the passage of Hurricanes Irma and María in September 2017, while codes marked with (+) indicate taxa detected only after the hurricanes. When available, Barcode Index Numbers (BINs) are included to support species identification and future reference via the Barcode of Life Data Systems (BOLD) platform.

**Species Code**	**Family**	**Subfamily**	**Species**	**BOLD BIN number**	**Number of individuals**		
					**P**	**T**	**Total**
K045(+)	Alucitidae		*Alucita* sp1		3	1	4
A182	Attevidae		*Atteva pustulella*	AAA9259	14	1	15
A208	Blastobasidae	Blastobasinae	*Blastobasis* sp1	ADE2065	4	2	6
K122(+)			*Blastobasis* sp2		3	4	7
K119(+)			*Holcocera* sp1		0	5	5
G015(+)	Cossidae	Zeuzerinae	*Psychonoctua muricolora*	AAH7923	0	2	2
A125			*Psychonoctua personalis*	AEM3584	16	50	66
A188	Crambidae	Acentropinae	*Chrysendeton anicitalis*	AEM0169	25	52	77
K137(+)			*Neargyractis plusialis*		1	0	1
M094			*Petrophila albulalis*	AEM0939	144	118	262
G001(+)			*Petrophila* sp1	ADG2045	0	2	2
M087			*Usingeriessa onyxalis*	AEM0940	117	97	214
A191(+)		Crambinae	*Argyria diplomochalis*	AAC0264	2	1	3
A218			*Crambus quinquareatus*	AEM0320	3	0	3
A213(–)			*Diatraea* sp1		1	0	1
A248(+)			*Fissicrambus profanellus*	AAA0300	1	0	1
A216			*Microcrambus biguttellus*	AEM0315	8	19	27
G007(–)		Evergestinae	*Trischistognatha* nr. *pyrenealis*	AAA0260	1	0	1
K088(+)		Glaphyriinae	*Dicymolomia metalophota*		0	1	1

M110			*Glaphyria badierana*	AEM3261	2	1	3
K144(+)			*Glaphyria dolatalis*		2	6	8
M048			*Psephis myrmidonalis*	AAG3083	23	51	74
M053			*Trischistognatha palindalis*	AEM2361	14	22	36
A016		Musotiminae	*Odilla noralis*		3	0	3
A184			*Undulambia rarissima*	AEM3961	1	4	5
A071			*Undulambia* sp1	AEM4802	1	6	7
A185		Pyraustinae	*Cryptobotys zoilusalis*	AAA0405	4	10	14
K008(+)			*Epicorsia cerata*		1	1	2
G003(+)			*Pyrausta* sp1	AEL9970	4	2	6
K098(+)		Schoenobiinae	*Donacaula* sp1		3	0	3
M007		Spilomelinae	*Apogeshna stenialis*	ADS0623, ADX4098	601	776	1377
M095			*Ategumia ebulealis*	AAA0471	10	25	35
A135			*Azochis rufidiscalis*	AEN2994	2	2	4
K010(+)			*Bradina hemmingalis*		3	4	7
K003(+)			*Ceratocilia* sp1		3	2	5
M003			*Condylorrhiza vestigialis*	AEL8590	22	67	89
A041			*Desmia julialis*	AEM1770	32	60	92
M002			*Desmia* nr. *ploralis* sp1	AAA0441	10	7	17
K011(+)			*Desmia* nr. *ploralis* sp2		0	2	2
A133(+)			*Desmia ufeus*	AAC2420	1	3	4
K138(+)			*Deuterophysa albilunalis*		12	19	31
M010			*Deuterophysa* sp1	AAF2121	35	45	80
A147(–)			*Diaphania elegans*	AAB7626	0	1	1
A084(+)			*Diaphania infimalis*	AAA0432	0	3	3
K005(+)			*Diaphania nitidalis*		0	1	1
K139(+)			*Diaphania ochrivitralis*		1	0	1
K009(+)			*Diaphantania candacalis*		0	2	2
K099(+)			*Diasemiopsis leodocusalis*		2	0	2
A190(+)			*Diasemiopsis ramburialis*	AAD0296	0	2	2
A194			*Diathrausta cubanalis*	AEM3588	5	9	14
A082			*Glyphodes sibillalis*	AAA0461	3	9	12
A253(+)			*Herpetogramma* nr. *aeglealis*	ACB9422	1	4	5
G008(+)			*Herpetogramma phaeopteralis*	ABY7602	1	5	6
A145(+)			*Herpetogramma semilaniata*	AAJ6265, AAA7564	0	10	10
A073			*Herpetogramma servalis*	AAE9273	21	62	83
A192(+)			*Herpetogramma* sp1	AAA0571	0	3	3
K007(+)			*Herpetogramma stramineata*		1	3	4
M008			*Hileithia ductalis*	ACN0621	15	24	39
A186			*Hymenia perspectalis*	AAA0344	5	13	18
A137(–)			*Lineodes* sp1	AEL8213	0	1	1
K140(+)			*Lygropia* sp1		2	5	7

A015			*Lygropia tripunctata*	AEM4195	4	27	31
G004(+)			*Marasmia* sp1	AAA0381	1	4	5
M005			*Maruca vitrata*	ADR7532	7	5	12
K074(+)			*Microphysetica hermeasalis*		10	12	22
A117			*Microthyris anormalis*	AAZ7619	2	7	9
A040			*Microthyris prolongalis*	AAA4646	45	52	97
A201			*Neurophyseta* sp1	AEL9990	13	13	26
K001(+)			*Omiodes humeralis*		5	3	8
A150			*Omiodes indicata*	AAB5389	9	10	19
K002(+)			*Omiodes martyralis*		0	1	1
A012			*Omiodes simialis*	AAM8859	2	2	4
M001			*Palpita isoscelalis*	AAF2122	12	74	86
G030(+)			*Palpita persimilis*	AAY8244	0	1	1
M055			*Palpita* sp1	AEM2053	31	36	67
G006(–)			*Palpita* sp2	AAN5523	0	1	1
A120(+)			*Palpusia goniopalpia*	AAB6536	1	0	1
K006(+)			*Pantographa limata*		1	1	2
A030			*Patania silicalis*	AAA0563	7	16	23
A265(+)			*Pilemia periusalis*	AAC6337	0	1	1
A081			*Pilocrocis ramentalis*	AAA0334	2	3	5
M025			*Polygrammodes elevata*	AAY2807	5	7	12
G005(+)			*Salbia abnormalis*	AAL8603	5	6	11
A141			*Salbia cassidalis*	AAB4416	191	758	949
M105			*Samea ecclesialis*	AAA0392	4	1	5
K004(+)			*Sathria onophasalis*		5	1	6
M077			*Sathria simmialis*	AEM2268	17	8	25
A049			*Sparagmia gonoptera*	AAB6753	2	9	11
A008			*Spilomela personalis*	AAC7836	3	4	7
A083(+)			*Synclera jarbusalis*	AAA0437	2	12	14
A177(–)			*Syngamia florella*	AAA0383	1	0	1
A139			Crambidae sp2	AAA4643	22	36	58
A198			Crambidae sp1	AEM3011	2	1	3
A193			Crambidae sp3	AEM3660	2	8	10
K043(+)	Depressariidae	Ethmiinae	*Ethmia confusella*		0	1	1
A199(–)			*Ethmia submissa*	ACS5943	1	0	1
A254(–)		Stenomatinae	*Mothonica ocellea*	AAA0941	2	0	2
K029(+)	Erebidae	Anobinae	*Deinopa ostia*		1	0	1
K058(+)		Arctiinae	*Afrida charientisma*		1	1	2
M029			*Correbidia terminalis*	AAA1326	10	12	22
A072(+)			*Cosmosoma auge*	AAE4005	3	4	7
A009			*Ctenuchidia virginalis*	AEM1882	10	15	25
K136(+)			*Eunomia colombina*		2	2	4
A068(+)			*Eupseudosoma involuta*	ABY6001	0	1	1
A111			*Halysidota ata*	ACD9303	4	2	6

A004			*Hyalurga vinosa*		3	4	7
A115(+)			*Hypercompe icasia*	ADW3484	1	1	2
A149			*Lomuna nigripuncta*	ACD9263	5	8	13
A010			*Lymire albipedalis*	ACD9338	1	3	4
A028			*Opharus bimaculata*	ACE7676	21	17	38
A132(–)			*Phoenicoprocta partheni*	ABW8788	1	0	1
A157			*Progona pallida*	ADE4311, AAH4992	861	904	1765
A136			*Tricypha proxima*	ACD9411	2	3	5
A168(–)			Erebidae sp2	AAH4983	3	4	7
A167		Boletobiinae	*Metalectra* sp1	AEM1772	8	13	21
A219(+)			*Mursa phtisialis*	AAG2981	0	1	1
A206(+)			*Ommatochila mundula*	ABZ6249	3	1	4
K017(+)		Calpinae	*Gonodonta incurva*		0	1	1
K016(+)			*Gonodonta sicheas*		1	1	2
A130(+)			*Parachabora abydas*	AAC7967	1	4	5
K015(+)			*Pararcte schneideriana*		2	0	2
M074			*Plusiodonta thomae*	AAF3702	2	3	5
A048(–)		Catocalinae	*Argidia sublevata*	AAA9964	0	1	1
K013(+)		Erebinae	*Ascalapha odorata*		1	0	1
K020(+)			*Euclystis deterrima*		0	1	1
A128(+)			*Mocis diffluens*	AAB7399	2	0	2
A144(+)			*Mocis disseverans*	AAA9789	0	1	1
A047(+)			*Mocis repanda*	ACD9194	0	1	1
M086			*Ophisma tropicalis*	AAA4625	8	7	15
K014(+)			*Thysania zenobia*		1	0	1
K019(+)			*Zale exhausta*		1	0	1
A076		Eulepidotinae	*Antiblemma sterope*	ABY4140	10	10	20
A014			*Anticarsia elegantula*	AEM1982	8	8	16
A052(–)			*Ephyrodes omicron*	AEM1884	2	1	3
A151(+)			*Eulepidotis addens*	ACE9114	1	0	1
K021(+)			*Eulepidotis* nr. *pavo*		4	2	6
K022(+)			*Eulepidotis superior*		1	0	1
A034(–)			*Metallata absumens*	AAC8148	1	0	1
G014(–)			*Metallata* nr. *absumens*	AEM4774	1	0	1
A160			*Renodes aequalis*	AEK9026	3	1	4
M070		Herminiinae	*Carteris oculatalis*	AAD5856	6	4	10
A252			*Heterogramma terminalis*	AEN3659	2	3	5
G019(+)			*Hypenula* sp1	AEY9316	1	1	2
A261			*Lascoria* nr. *aon*	AAI1279	5	4	9
A258(–)			*Lascoria* nr. *majoralis*	ACR3487	1	0	1
A215(+)			*Lascoria orneodalis*	AAB4990	1	1	2
A178			*Lascoria* sp1	AEL8619	4	6	10
A021			*Lophophora clanymoides*	ADE4020, AEN0351	66	93	159

K060(+)			*Mastigophorus demissalis*		1	4	5
A169(+)			*Phalaenophana* nr. *pyramusalis*	AAH3513	3	6	9
A119			*Phlyctaina irrigualis*	AAE0446	0	6	6
A019			*Physula albipunctilla*	AAH9380	37	49	86
G021			*Renia* sp1	ACD4536	2	2	4
K079(+)			*Tetanolita mutatalis*		1	1	2
A017			Erebidae sp3	AEL9527	22	71	93
K052(+)		Hypeninae	*Hypena philomedia*		0	1	1
A251(+)			*Hypena porrectalis*	AAB3497	1	3	4
A245(+)		Hypenodinae	*Hypenopsis calusa*	AAB2278	1	3	4
K096(+)			*Hypenopsis* sp1		1	3	4
A045		Omopterini	*Zale peruncta*	AAB1416	3	0	3
A250(–)		Ophiusini	*Selenisa portoricensis*	AAC4416	0	1	1
A239		Rivulinae	*Rivula pusilla*	AAB2943	48	54	102
K081(+)			Erebidae sp1		1	0	1
A242(+)	Gelechiidae	Dichomeridinae	*Dichomeris* nr. *costalis*	AEM2170	1	1	2
G012(–)		Gelechiinae	*Symmetrischema* sp1	ADG1165	0	1	1
K053(+)			Gelechiidae sp1		0	1	1
M017	Geometridae	Desmobathrinae	*Leptoctenopsis* sp1	AEL9609	14	21	35
A065		Ennominae	*Covellia* sp1	AEM3945	1	1	2
A223(+)			*Cyclomia* sp1	AEM3809	2	0	2
M013(–)			*Epimecis jamaicaria*	AEL9097	15	5	20
A038			*Macaria* nr. *regulata*	AAF9507	3	10	13
A171(+)			*Macaria* sp1	ADJ0673	2	0	2
A086			*Oxydia vesulia*	AAA5961	22	22	44
A099			*Patalene hamulata*	AAA7956	20	13	33
A070(–)			*Pero rectisectaria*	ACU3922	1	0	1
A027(–)			*Phrygionis moeschleri*	ACQ5941	2	1	3
A172(+)			*Psamatodes* nr. *abydata*	AEM4464	1	0	1
A044			*Pyrinia sanitaria*	ACU2958	2	5	7
M015			*Sabulodes curta*	AEM0209	6	9	15
A126(+)			*Semiothisa* sp1	AEM1975	0	1	1
K023(+)		Geometrinae	*Chloropteryx* sp1		1	1	2
A007			*Phrudocentra* nr. *centrifugaria*	AEM1154	10	2	12
A051			*Synchlora cupedinaria*	AEM0864	10	15	25
A031			*Synchlora* nr. *herbaria*	AEM1799	11	37	48
A006			*Synchlora* nr. *xysteraria*	AEM1917	23	13	36
M080		Larentiinae	*Eois snellenaria*	AEM1432	9	7	16
A246(–)			*Eupithecia* sp1	AEM3808	2	0	2
K100(+)			*Eupithecia* sp2		1	0	1
K030(+)			*Eupithecia velutipennis*		5	9	14
A220(+)			*Psaliodes* sp1	AEM2771	2	0	2
M106			*Psaliodes* sp2	AEM3810	5	7	12

K048(+)			Geometridae sp1		2	1	3
A054		Sterrhinae	*Cyclophora* nr. *rubrior*	AEN1135	1	2	3
M019(–)			*Cyclophora* sp1	AEM3583	9	12	21
M026			*Idaea monata*	AEM3582, ADR5174	67	141	208
K027(+)			*Leptostales praepeditaria*		4	2	6
A138(+)			*Lobocleta* nr. *tenellata*	AEM0454	0	8	8
A058			*Pleuroprucha* nr. *molitaria*	ADE2782	22	20	42
G010(–)			*Pleuroprucha rudimentaria*	AAB9656	9	1	10
A033			*Semaeopus* sp1	AEM4664	3	6	9
A011			*Tricentrogyna* sp1	AEM0377	8	16	24
M079			*Tricentrogyna vinacea*	AEL7533	22	35	57
M081	Limacodidae		*Heuretes picticornis*	ADB4913	11	1	12
M066	Megalopygidae	Megalopyginae	*Megalopyge krugii*	AEZ7402	23	8	31
K072(+)	Noctuidae	Amphipyrinae	*Anateinoma affabilis*		0	3	3
A127(+)		Condicinae	*Condica cupentia*	AAA7985	1	0	1
A075(–)			*Condica funerea*	AAB9353	1	0	1
K085(+)			*Condica punctifera*		0	1	1
A124(+)			*Condica sutor*	AAA5860	0	1	1
A142(+)		Eriopinae	*Callopistria floridensis*	AAA4051	1	0	1
A152(+)		Eustrotiinae	*Marimatha* sp1	ABZ5505	0	2	2
A243(+)			*Ozarba* nr. *semipotentia*	AEL8774	3	1	4
K032(+)			*Tripudia* nr. *luda*		1	3	4
K067(+)		Noctuinae	*Acroria pulchra*		0	1	1
A043(–)			*Dargida soligena*	AEM3837	2	1	3
A158			*Dypterygia ordinarius*	AAA5250	1	2	3
G018(+)			*Elaphria agrotina*	AAA4470	0	2	2
G024(+)			*Elaphria deltoides*	AAU0963	1	0	1
K083(+)			*Elaphria guttula*		1	0	1
A118			*Gonodes* nr. *liquida*	AEL8696	1	5	6
A069(–)			*Marilopteryx* sp1	AEL8214	1	0	1
M050			*Paratrachea spangleri*	ACD9199	27	33	60
A116(+)			*Spodoptera eridania*	AAA6521	0	9	9
A074(+)			*Spodoptera frugiperda*	AAA4532	1	2	3
K068(+)			Noctuidae sp3		0	1	1
A143(+)		Plusiinae	*Argyrogramma verruca*	AAC5989	0	1	1
K090(+)	Nolidae	Chloephorinae	*Garella nilotica*		2	0	2
A129(+)		Collomeninae	*Collomena filifera*	AAC8703	0	2	2
A140(–)			*Gyrtonides albifascia*	AEM1871	1	0	1
A123		Nolinae	*Nola bistriga*	AEM4057	76	143	219
K112(+)			*Nola* sp1		0	1	1
A003	Notodontidae	Heterocampinae	*Cecrita* nr. *proba*	AEM3248	14	23	37
K143(+)			*Disphragisella baracoana*		1	0	1
A059(+)			*Ginaldia distinguenda*	AAA6354	1	7	8
A001(–)		Nystaleinae	*Nystalea superciliosa*	AAA8476	2	1	3

K120(+)	Pterophoridae	Pterophorinae	Pterophoridae sp1		1	0	1
A077(+)	Pyralidae	Chrysauginae	*Bonchis munitalis*	ACY7305	1	5	6
M052			*Caphys bilineata*	AEL8840	45	195	240
M096			*Murgisca subductellus*	ADS4752	5	9	14
A211(–)			*Salobrena rubiginea*	ACU7252	1	0	1
K033(+)			*Streptopalpia minusculalis*		4	13	17
K047(+)			*Tosale oviplagalis*		7	2	9
A134		Epipaschiinae	*Dasyvesica nepomuca*	AAH5005	4	15	19
A078			*Deuterollyta majuscula*	AEM1298	10	9	19
A032			*Deuterollyta ragonoti*	AEM1448	33	71	104
A209			*Phidotricha erigens*	AAA0929	2	2	4
M100(–)			*Pococera scabridella*		0	1	1
A155			*Pococera* sp1	AEM2065	2	5	7
G031(–)			Pyralidae sp2		0	1	1
K095(+)		Galleriinae	*Achroia grisella*		0	1	1
A153			*Alpheias* sp1	AEL7770	8	50	58
A056			*Galleria mellonella*	AAA0965	5	4	9
K113(+)			*Genopaschia protomis*		1	1	2
K101(+)			*Pogrima palmasalis*		2	0	2
K116(+)			Pyralidae sp3		1	0	1
A210(–)		Phycitinae	*Anegcephalesis arctella*	ADK6931	0	1	1
G032(–)			*Atheloca subrufella*	AAG0404	1	0	1
G011(–)			*Coptarthria dasypyga*	AEA3910	1	0	1
A025			*Davara caricae*	AAA5501	36	69	105
A163			*Davara interjecta*	AEM2964	25	46	71
M104			*Hypargyria definitella*	AAA5507	102	27	129
A205(+)			*Hypsipyla grandella*	AAA5499	1	1	2
A154			*Megarthria petersoni*	ABW7510	4	7	11
K133(+)			*Mescinia* nr. *parvula*		4	3	7
A259			*Nonia exiguella*	AEN5307	0	3	3
A162			*Oryctometopia fossulatella*	AAA5505	1	10	11
A249			*Piesmopoda apocerastes*	ADU7995	12	20	32
M045			*Piesmopoda flavicans*	AED1684	38	55	93
A237(+)			Pyralidae sp4	AEL9649	0	4	4
K110(+)			Pyralidae sp5		0	1	1
K141(+)			Pyralidae sp6		0	1	1
G002(+)		Pyralinae	*Hypsopygia nostralis*	AAI3521	0	3	3
G009(+)			Pyralidae sp1	ACU7183	0	1	1
A046	Sphingidae	Macroglossinae	*Perigonia lusca lusca*		1	3	4
A112(+)			*Xylophanes pluto*	AAA4190	0	1	1
A042(–)		Sphinginae	*Cocytius duponchel*	AAA3938	1	0	1
M085			*Manduca brontes pamphilius*	ADW3128	7	3	10
A200	Thyrididae	Siculodinae	*Rhodoneura thiastoralis*	AEM3309	37	68	105
K041(+)			*Zeuzerodes maculata*		0	3	3

A005		Striglininae	*Banisia furva*	AEL9849	8	13	21
A131	Tineidae	Acrolophinae	*Acrolophus* nr. *ochracea*	AEL9768	1	1	2
A159			*Acrolophus* sp1	ACD9349	3	1	4
A179			*Acrolophus* sp2	ADE6328	4	3	7
A207			*Acrolophus* sp3	AEM3070	11	6	17
A230(+)			*Acrolophus* sp4		0	1	1
A240(+)			*Acrolophus* sp5	ADE8398	11	11	22
A260(+)			*Acrolophus* sp6	AEM8189	1	1	2
K039(+)			*Acrolophus* sp7		0	1	1
K056(+)			*Acrolophus* sp8		1	2	3
A196			*Amydria* sp1	AEM1699	8	1	9
K091(+)			*Amydria* sp2		1	0	1
K142(+)			*Amydria* sp3		0	1	1
K121(+)			Tineidae sp2		1	0	1
A122		Hapsiferinae	*Tiquadra* nr. *avitella*	AEL7342	1	4	5
G026(–)		Scardiinae	*Xylesthia* sp1	AEZ0099	1	0	1
G013(+)			*Lepyrotica* sp1	AEN1138	1	0	1
A238(+)			Tineidae sp1	AEM0842	1	0	1
A202(–)	Tortricidae	Chlidanotinae	*Heppnerographa tricesimana*		0	1	1
A203(+)		Olethreutinae	*Bactra philocherda*	AAA0293	37	22	59
K037(+)			*Cacocharis albimacula*		0	1	1
G033(–)			*Crocidosema longipalpana*	ADE8754	1	0	1
A256			*Crocidosema* nr. *lantana*	ADS1687	10	15	25
A262			*Cryptaspasma bipenicilla*	ACH2101	6	3	9
A247(+)			*Episimus* nr. *rufatus*	AEM3811	0	2	2
A241(–)			*Episimus* sp1	AEL8652	2	0	2
M109(–)			*Episimus* sp2		1	0	1
A148(+)			*Gymnandrosoma leucothorax*	ACH2052	1	1	2
A212			*Gymnandrosoma trachycerus*		0	3	3
G035(+)			*Rhopobota* sp1	AEY8134	0	1	1
G023			*Strepsicrates* sp1	AAA0955	1	4	5
G036(–)			Tortricidae sp1		1	0	1
G034(–)			Tortricidae sp2		0	1	1
K059(+)		Tortricinae	*Coelostathma* nr. *parallelana*		0	1	1
A024(+)			*Phricanthes* sp1	AEM0542	0	1	1
K094(+)			Tortricidae sp3		1	0	1
A121(+)	Uraniidae	Epipleminae	*Antiplecta* nr. *triangularis*	AAD7314	5	4	9
A079	Unknown		Unknown sp1	AEM0218, AEM0219	21	21	42
A263			Unknown sp2	AEN1816	1	1	2
Discarded					94	79	173
			**Total (including Discarded)**		**3932**	**5703**	**9635**
			**Total (excluding Discarded)**		**3838**	**5624**	**9462**
